# A three-year audit of pregnancy outcomes in women with pulmonary hypertension admitted to the high-risk obstetric unit at Inkosi Albert Luthuli Central Hospital, KwaZulu-Natal, South Africa

**DOI:** 10.5830/CVJA-2022-061

**Published:** 2022-12-05

**Authors:** S Budhram, P Krishundutt

**Affiliations:** Department of Obstetrics and Gynaecology, Tygerberg Hospital, and Faculty of Medicine and Health Sciences, Stellenbosch University, Stellenbosch, South Africa; Discipline of Obstetrics and Gynaecology, School of Clinical Medicine, College of Health Sciences, University of KwaZulu-Natal, Durban, South Africa

**Keywords:** pregnancy, pulmonary hypertension

## Abstract

**Objective:**

The aim of this study was to describe the profile and outcomes of pregnancies in women with pulmonary hypertension in South Africa.

**Methods:**

A retrospective study was undertaken at a statesubsidised hospital. Data were analysed using SPSS. Descriptive statistics were used to summarise categorical variables. Central tendency and dispersion of data were measured using means and standard deviations for normally distributed variables and medians and interquartile ranges for skewed variables. A p-value less than 0.05 was considered statistically significant.

**Results:**

Of a cohort of 185 women, 86.3% had pulmonary hypertension secondary to left heart disease. The median age of the cohort was 28 years (interquartile range 23–33) with 37.8% being HIV infected and 59% having mild pulmonary hypertension. Frequencies of deaths, intensive care unit admissions and cardiac failure events increased with increasing severity of pulmonary hypertension (p < 0.001). Women with more severe pulmonary hypertension had higher rates of preterm births (p < 0.001).

**Conclusion:**

Adverse pregnancy outcomes were concentrated in women with moderate-to-severe pulmonary hypertension.

 Pulmonary hypertension (PH) is a rare yet important condition that may complicate pregnancy. The literature, mostly dating back to the 1990s, reports maternal mortality rates of between 30 and 56% in women with PH.^1^ Poor perinatal outcomes have also been reported, with high rates of preterm delivery, foetal growth restriction, stillbirth and neonatal death.^1^-^3^

 It is thought that the haemodynamic, anatomical and biochemical changes that accompany pregnancy, delivery and the puerperium render pregnant women less tolerant to the effects of PH,^4^ predisposing them to morbidity and mortality. Hence it is recommended that pregnancy be avoided in these women, and if it occurs, then a termination should be discussed.^5^

PH is categorised into five groups based on aetiology: pulmonary artery hypertension (PAH), PH due to left heart disease, PH due to lung diseases and/or hypoxia, chronic thromboembolic PH, and PH with unclear multifactorial mechanisms.^6^

 The literature on pregnancy courses and outcomes in women with PH is limited. A recent international registry reported outcomes of pregnancy in women with PH and showed a muchimproved mortality rate of less than 5% for their entire cohort of 151 women.^7^ This cohort was made up of women from both high-, and low- and middle-income countries.

 There is a large disparity in maternal and perinatal morbidity and mortality rates across the globe, with poorer countries bearing greater burdens of adverse outcome for various reasons. The most recent statistics from the World Health Organisation reported the maternal mortality rate in developed economies to be around 12 per 100 000 live births (0.012%), and 239 per 100 000 live births (0.2%) in emerging economies, with large disparities both between and within countries.^8^

 It would therefore be premature to extrapolate findings from the cited study^7^ to a South African state-subsidised hospital setting. We have chosen to define our population and describe pregnancy courses and outcomes of women with PH in a South African state-subsidised hospital setting to assist with patient information and counselling.

## Methods

This descriptive, retrospective chart review was conducted at the high-risk obstetric unit (HROU) at Inkosi Albert Luthuli Central Hospital (IALCH) in KwaZulu-Natal (KZN), South Africa, following ethical approval from the University of KwaZulu- Natal’s biomedical research ethics committee (BE 413/18).

The HROU at IALCH is a central referral unit providing quaternary-level care to all women at high risk for adverse maternal and/or foetal outcomes. Medical records at IALCH are stored electronically in the hospital information system, Meditec. ICD 10 codes were used to retrieve consecutive records of all women with a diagnosis of PH admitted to the HROU from 1 January 2016 to 31 December 2018.

 Retrieved records were subsequently reviewed for eligibility to enter the study as per inclusion and exclusion criteria. The inclusion criterion was records of all pregnant women admitted with PH. Exclusion criteria were records of women without an echocardiogram documenting pulmonary artery pressure (PAP) and those with multiple pregnancies.

 Each patient was allocated a unique study number for the purposes of data collection and to maintain patient confidentiality. Data were initially collected on a pre-designed data-collection form, verified, electronically captured on Excel 2019 (Microsoft, USA) and analysed using SPSS version 25.0 (IBM Corporated, USA).

 The echocardiograms reported a mean PAP (mPAP). This was determined as follows: Doppler echo was used to determine the systolic PAP by measuring the flow velocity over the tricuspid valve and converting it to mPAP using Bernoulli’s equation. PH was defined as mPAP of ≥ 25 mmHg. Mild PH was defined as a resting mPAP ≥ 25 mmHg and < 50 mmHg. Moderate PH was a resting mPAP from 50 to 89 mmHg. Severe PH was a resting mPAP ≥ 90 mmHg.

 Maternal death was defined as death from any cause related to or aggravated by pregnancy or its management (excluding accidental or incidental causes) during pregnancy and childbirth or within 42 days of termination of pregnancy, irrespective of the duration and site of the pregnancy.

 Intrauterine growth restriction was defined as foetal abdominal circumference and/or expected foetal weight below the 10th percentile for gestational age, associated with an abnormal umbilical artery resistance index. Low birthweight was defined as a birth weight of less than 2 500 g.

 Preterm delivery was defined as any birth before 37 weeks and zero days of pregnancy. Late preterm delivery was any birth at a gestational age between 34 weeks and zero days, and 36 weeks and six days. Early preterm delivery was any birth at a gestational age between 24 weeks and zero days, and 33 weeks and six days.

## Statistical analysis

Continuous variables, such as patient ages, were summarised as mean ± standard deviation or median and interquartile range (IQR) as appropriate, and compared using the Student’s t-test or Wilcoxon–Mann–Whitney test as appropriate. Categorical variables, such as co-morbidities, were summarised as percentages and proportions and compared using the chi-squared test or Fisher’s exact test, as appropriate. A one-way ANOVA was used to compare patient outcomes among groups of different PAP. The level of statistical significance was set at a p-value of < 0.05.

## Results

**Fig. 1:**
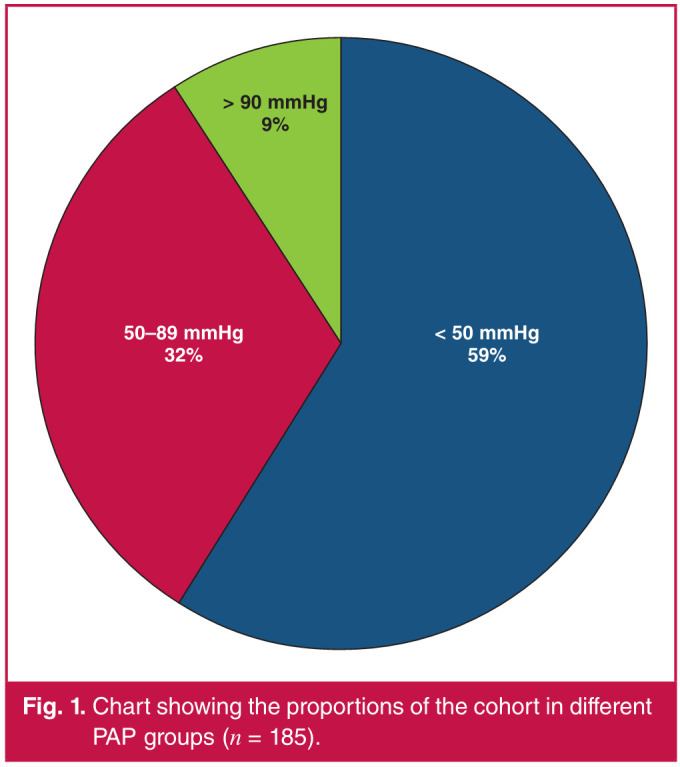
Chart showing the proportions of the cohort in different PAP groups (n = 185).

There was a total of 185 women with PH admitted to the HROU for the study period. The median (IQR) age of the cohort was 28 (23–33) years, with approximately 38% of the cohort being human immunodeficiency virus (HIV) infected at the first visit. Further analysis of baseline characteristics of the cohort, categorised according to PAP, showed no statistical differences among the categories, or in the prevalence of HIV infection (Table 1). The majority of women [109 (59%)] in the cohort had mild PH (Fig. 1).

**Table 1:** Characteristics of cohort at admission

*Characteristics*	*Entire cohort (n=185)*	*PAP <50 mmHg (n 109)*	*PAP 50-89 mmHg (n =60*	*PAP > 90 mmHg (n= 16)*	p-value
Age (years), median (IQR)	28 (23-33)	26 (22-33)	30 (23-33)	27 (26-33)	
Parity, median (IQR)	1 (0-2)	1 (0-1)	1 (0-2)	1 (0-2)	
HIV status, n (%)					
Non-infected	115 (62.2)	75 (65.2)	32(27.8)	8 (7.0)	
Infected	70 (37.8)	34 (48.6)	28 (40.0)	8 (11.4)	0.08

**Table 2:** Classification of PH in cohort (n = 185)

*Groups*	*Number of women*	*Percentage of entire cohort*
Group 1	24	13.7
Idiopathic	8	4.5
Associated with:		
Connective tissue disease	2	1.1
Congenital heart disease		
Patent ductus arteriosus	2	1.1
Tetralogy of Fallot	4	2.3
Ventricular septal defect	4	2.3
Atrial septal defect	2	1.1
Transposition of great arteries	1	0.6
Ebstein's anomaly	1	0.6
Group 2	151	86.3
Mitral stenosis	33	18.9
Mitral regurgitation	28	16
Mixed mitral valve disease	38	21.7
Aortic valve disease	9	5.1
Mitral and aortic valve disease	1	0.6
Tricuspid regurgitation	4	2.3
Cardiomyopathies	9	5.1
Prosthetic valves	25	14.3
Other	4	2.3

Classification of the cohort according to the cause of PH showed that most women [151 (85%)] had PH secondary to acquired heart disease, and within this group, the leading cause (21.7%) was mixed mitral valve disease. Group 1 (PAH) accounted for 13.7% of the cohort (Table 2). PH of other causes (PH due to lung disease, PH due to pulmonary artery obstruction and PH with multifactorial/unclear mechanisms) was not present in the cohort.

**Fig. 2:**
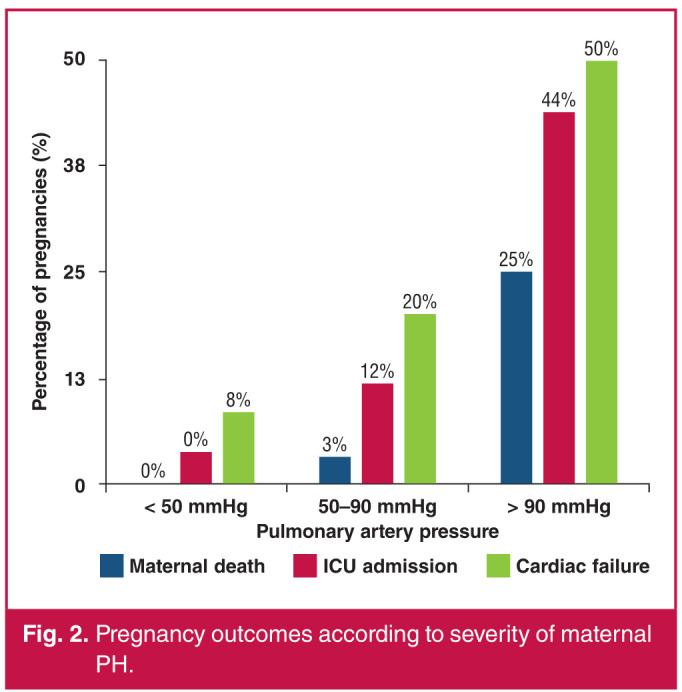
Pregnancy outcomes according to severity of maternal PH.

Maternal outcomes were significantly different among the PAP groups (Fig. 2), with approximately eight, 20 and 50% of women in the mild, moderate and severe categories of PH, respectively, experiencing one or more episodes of cardiac failure during the pregnancy or puerperium (p < 0.001). The number of women admitted to an intensive care unit (ICU) also increased significantly with increasing PAP (p < 0.001), with more than 40% of women in the severe PH category needing admission to an ICU. There were five maternal deaths in the entire cohort, of whom three women had severe PH and two had moderate PH (p < 0.001). A detailed description of the maternal deaths is presented in Table 3.

**Table 3:** Maternal deaths (n = 185)

*Deaths*	*< 50 mmHg*	*50-89 mmHg*	*90 mmHg*	Total
Alive beyond 42 days, n	109	58	13	179
Maternal death, n (%)	0	2 (3.33)	3 (18.75)	5
Total	109	60	16	185

Of the 185 women in the cohort, there were four who underwent elective medical terminations of their pregnancies and three who experienced spontaneous miscarriages (< 24 weeks). There were 109 women in the group with mild PH, and of these, one underwent elective medical termination of pregnancy and two had miscarriages. Twenty-two per cent of these women had a normal vaginal delivery while 75.3% had a caesarean section (C/S) (30.3% were elective C/S deliveries and 45% were emergency C/S deliveries). This was 58.6% (82/140) of the total number of C/S deliveries.

 There were 60 women in the group with moderate PH, and of these, two had elective medical terminations of pregnancy and one had a miscarriage. Twenty per cent of these women had a normal vaginal delivery while 75% had a C/S (48.3% were elective C/S deliveries and 26.7% were emergency C/S deliveries). This was 32.1% (45/140) of the total number of C/S deliveries.

**Fig. 3:**
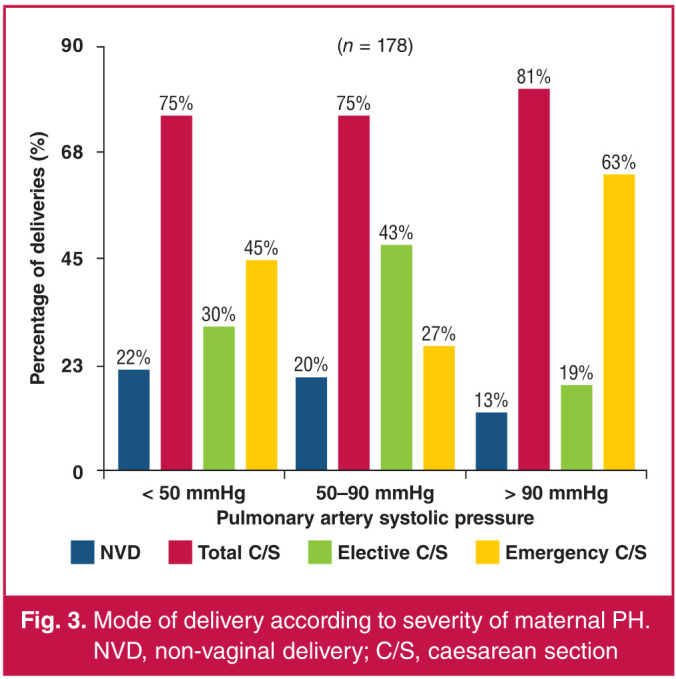
Mode of delivery according to severity of maternal PH. NVD, non-vaginal delivery; C/S, caesarean section

There were 16 women in the group with severe PH, and of these, one had an elective termination of pregnancy and there were no miscarriages; 12.5% of these women had a normal vaginal delivery while 81.3% had C/S deliveries (18.75% were elective C/S deliveries and 62.5% were emergency C/S deliveries. This was 9.3% (13/140) of the total number of C/S deliveries. The overall C/S rate was 78.7%. Elective C/S deliveries accounted for 46.4% (65/140) and emergency C/S deliveries accounted for 53.6% (75/140) (Fig. 3).

**Fig. 4:**
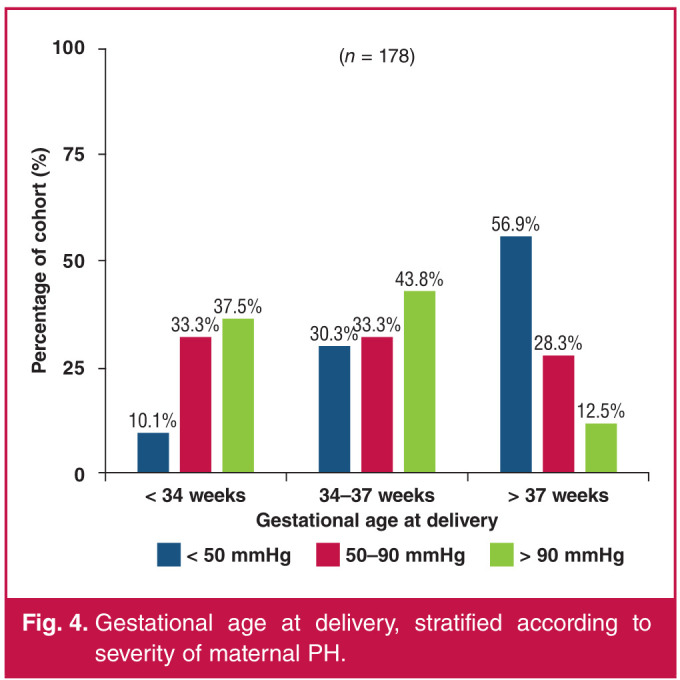
Gestational age at delivery, stratified according to severity of maternal PH.

 Among women who had early preterm (< 34 weeks) and late preterm (34–37 weeks) deliveries (n = 97), the majority (37.5 and 43.8%, respectively) were in the group of women with severe PH and the minority (10.1 and 30.3%, respectively) in the group of women with mild PH. Among those who delivered beyond 37 weeks of gestation (n = 81), there were 56.9% in the mild PH group and 12.5% in the severe PH group (p < 0.001). In total, 54.5% (97/178) of women delivered before 37 weeks of gestation and 45.5% (81/178) delivered beyond 37 weeks of gestation (Fig. 4).

 There were six stillbirths in the cohort, resulting in a foetal loss rate of 3.4%. This left 172 live births in which the birth characteristics were analysed. With increasing severity of PH, the proportions of growth-restricted foetuses increased by 7.8, 17.9 and 28.6% in the categories of mild, moderate and severe PH, respectively, with a total of 12.8% (22/172) of foetuses being growth restricted (p = 0.036).

**Fig. 5:**
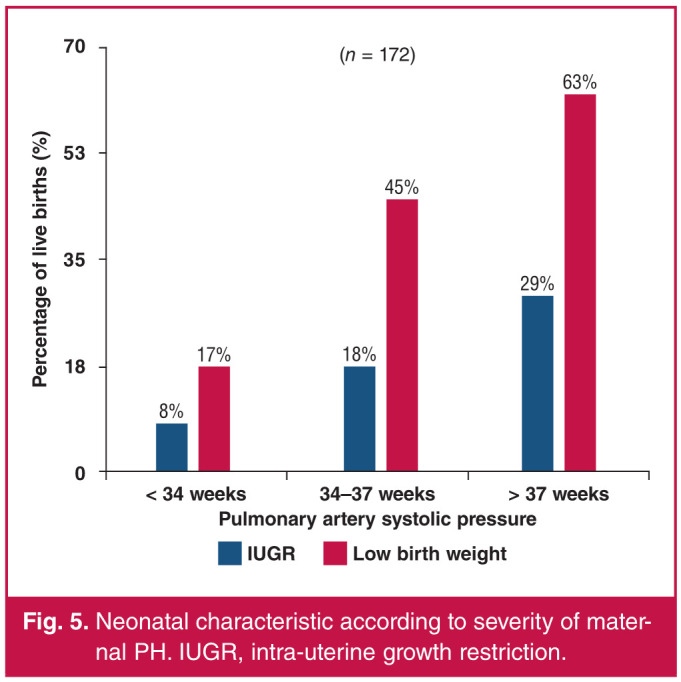
Neonatal characteristic according to severity of maternal PH. IUGR, intra-uterine growth restriction.

Similarly, as the PH increased in severity, the proportion of low-birthweight (< 2 500 g) babies born increased by 17.4, 45 and 62.5% in the mild, moderate and severe PH groups, respectively. A total of 30.2% (52/172) of foetuses had low birth weight (p < 0.001) (Fig. 5).

 There were five maternal deaths (up to 42 days postpartum) in the cohort. Two (3.3%) of the maternal deaths were in the moderate PH group and three (18.8%) were in the severe PH group (Table 3). An additional death of a woman with severe PH was recorded three months postpartum (admitted with cardiac failure and requiring urgent mitral valve replacement) but this was not analysed for statistical purposes. Of the entire cohort, 2.7% of women suffered a mortality (Table 4).

**Table 4:** Description of maternal deaths

*Patient*	*Age (years)*	*Diagnosis*	*Parity*	*Gestational age at 1st visit (weeks)*	*PAP (mmHg)*	*Complications*	*Gestational age at delivery (weeks)*	*Delivery*	*Foetal state*	*Time of maternal death*	Cause of death	Additional information
1	26	Mitral stenosis (MVA: 0.58 cm²) diagnosed in pregnancy	G1P0	19	115	Cardiac failure	30	Emergency C/S for foetal compromise	Alive	Day 1 post C/S	Cardiac failure	Decompensated intra-op post delivery - died day 1 post delivery in ICU
2	39	Asthma/ obesity/ peripartum cardiomyopathy X 4	G5P4	31	51	Septicaemia/ cellulitis/ renal failure	34	NVD	Alive	Day 3 post NVD	Cardiac failure	Cardiac failure secondary to cardiomyopathy - died in ICU, was on ionotropic support
3	22	MMVD (MVA: 1.24 cm2) diagnosed in pregnancy	GIPO	27	52	Developed cardiac failure/ severe anaemia post delivery	37	Elective C/S for cardiac disease	Alive	Day 30 post C/S	Cardiac failure	
4	28	TB pericarditis (treated 4 years prior to pregnancy)	G3P1	20	110	Cardiac failure/sepsis	37	Emergency C/S for worsening cardiac failure	Alive	Day 2 post C/S	Cardiac failure	Developed cardio- respiratory failure and sepsis peri- delivery
5	30	Severe tricuspid regurgitation (diagnosed in pregnancy)	G2P1	37	91	Cardiac failure	37	Emergency C/S for worsening cardiac failure	Alive	Day 2 post C/S	Cardiac failure	Transferred to ICU from peripheral hospital with cardiac failure and newly diagnosed severe tricuspid regurgitation with pulmonary hypertension

MVA, mitral valve AREA; MMVD, mixed mitral valve disease; C/S, caesarean section; G, gravidity; P, parity; ICU, intensive care unit.

## Discussion

This report of our cohort of 185 women, admitted with PH during pregnancy, is the largest of its kind published in South Africa and possibly globally. This large sample size contributes to the reliability and relevance of the study findings. The largest international study cohort, prior to ours, was that by Sliwa et al., consisting of 151 women with PH, and locally by Osman et al., consisting of 52 women with PH.^7^,^9^

 HIV-infected women made up 37.8% of our cohort. According to the 2017 National Antenatal Sentinel HIV survey, the overall prevalence of HIV infection among antenatal clinic attendees in South Africa was 30.7%, with KZN having the highest prevalence of 41.1%.10 This provides some reassurance that our cohort was representative of the population of pregnant women in KZN.

 The Heart of Soweto study, conducted in Gauteng, South Africa, showed an incidence of new-onset rheumatic heart disease of 23.5/100 000 per annum among patients aged more than 14 years.^11^ Echocardiographic surveillance of South African school-aged children showed a prevalence of rheumatic heart disease of 20.2/1 000.12 In our cohort, the majority of women (86.3%) had diagnoses of PH secondary to left heart disease, classified as group 2 in the most recently updated classification of PH,^6^ largely caused by acquired mitral valve disease. This finding is in keeping with the large burden of rheumatic heart disease found in South Africa, as group 2 encompasses acquired vulvar lesions, including rheumatic heart disease. For this and other reasons, including socio-economic and cultural, the findings of many internationally published studies may not be applicable to the South African population.

 The ROPAC study (2016),^7^ originating from a multinational registry with South Africa as a contributor, and the only other local study,^9^ conducted in the Western Cape, South Africa, are the only two cohorts comparable to ours, where most women had diagnoses of PH belonging to group 2 and suffered from PH secondary to valvular heart disease. Additionally, the minority of women in these studies suffered from a severe form of PH, as was the case in our cohort. This is in stark contrast to the systematic review published by Jha et al. in 2020, which sought to summarise the outcomes of pregnant women with PH over the last three decades. In their review, most women had diagnoses of PH secondary to congenital heart disease. This consisted mostly of women with isolated atrial septal defects (19.4%) and ventricular septal defects (18.9%).^13^

 With regard to maternal outcomes, PH in pregnancy can lead to cardiac failure, which accounts for much of the morbidity and mortality associated with this diagnosis.

 The overall incidence of cardiac failure in our cohort was just under 16%. The incidence of cardiac failure increased proportionally to the severity of PH, with 8.6, 20 and 50% of women with mild, moderate and severe PH, respectively, experiencing one or more episodes of cardiac failure (p < 0.001).

The ROPAC cohort showed a similar relationship with rising PAP and increasing incidence of cardiac failure. Additionally, the indication for admission to hospital in the majority of women in their cohort was a diagnosis of cardiac failure. The overall incidence of cardiac failure in the ROPAC cohort was 20.5%.7 The Western Cape study, on the contrary, demonstrated a very high incidence of cardiac failure of 88%, with no relationship between the severity of PH and the incidence of cardiac failure, which may need to be investigated further.^9^

 In our cohort, the need for admission to an ICU mirrored that of the incidence of cardiac failure, with most admissions to ICU occurring in women with severe PH and the fewest admissions occurring in women with mild PH (p < 0.001). Other studies did not report on the need for ICU admission in their cohorts, although one would expect it to follow the pattern of cardiac failure.

Maternal mortality in women with PH is a grave concern, faced throughout pregnancy and the puerperium by women and their healthcare providers alike. In our cohort, there were no deaths in the group of women with mild PH, two deaths in the group with moderate PH and three deaths in the group with severe PH (p < 0.001). All deaths occurred in the postpartum period with four out of five deaths occurring in the first week postpartum. In the ROPAC cohort, there were five deaths up to 42 days postpartum; one of a woman with mild PH, three with moderate PH and one with severe PH and all deaths occurred in the first week postpartum.^7^ There was one death up to 42 days postpartum, reported in the Western Cape study, which occurred at two weeks postpartum.^9^

 Our case fatality rate up to 42 days postpartum was 2.7%. This rate is comparable to that of the ROPAC and Western Cape studies, which reported 3.3 and 1.9%, respectively.^7^,^9^ The systematic review published by Jha et al. in 2020 reported a slightly higher maternal mortality rate of 11.5 per 100 pregnancies.^13^ Overall, these rates are much lower than that reported in the older literature, which ranged between 25 and 56%.8,^14^

 The improvement in mortality rate may be attributable to many factors, including but not limited to, adoption of multidisciplinary team management strategies and improvements in therapeutics. Although mortality in women with PH in pregnancy remains a serious concern, there is some reassurance from our study and that of the studies cited above that the overall mortality rate has decreased significantly over time. Mortality is more likely to occur in women with more severe forms of PH and more emphasis needs to be placed on improving outcomes in the postpartum period, where the dramatic physiological changes associated with pregnancy appear to have a significant negative impact on the pathology associated with PH.

 In our cohort of 185 women, four underwent elective medical terminations of their pregnancies and three experienced spontaneous miscarriages before 24 weeks, leaving reportable delivery outcomes for 178 women.

 C/S was the predominant mode of delivery in our cohort, accounting for close to 80% (n = 140) of all deliveries, with 46.4% (65/140) being elective and 53.6% (75/140) being emergency procedures. Within each category of PH severity, C/S remained the predominant mode of delivery. The proportion of C/S deliveries in our cohort appears to be high in comparison to the ROPAC cohort (C/S deliveries in 63% of the cohort) and the Western Cape cohort (C/S deliveries in 54% of the cohort).7,^9^ In our cohort, further data would need to be collected pertaining to the indications for C/S deliveries to elucidate the seemingly high proportion of C/S deliveries.

 With regard to foetal and neonatal outcomes, analysis of gestational age at delivery in our study showed it to be inversely proportional to the severity of PH in the mothers. Women with more severe disease delivered more remotely from term as opposed to those with milder disease, who mostly delivered at term gestational age. The overall proportion of women who delivered preterm (< 37 weeks) was 54.5% (n = 97), which was made up of 38.1% (37/97) of women with an early preterm (< 34 weeks) delivery and 61.9% (60/97) with a late preterm (34–37 weeks) delivery. The ROPAC and Western Cape studies7,^9^ did not report on preterm deliveries. The systematic review by Jha et al. reported a preterm delivery rate of 51.7 per 100 deliveries in their review.^13^ More data are needed to support our findings but it is useful to know that women with milder forms of PH are likely to carry pregnancies closer to term and that their offspring are less likely to suffer from the complication of prematurity.

 There were six stillbirths in our cohort, leaving 172 live births in which the birth characteristics were analysed. Overall, there were 12.8 and 30.2% of growth-restricted and low-birthweight babies, respectively, in our cohort. The severity of PH was proportional to the number of growth-restricted neonates, with the majority occurring in women with severe PH (p = 0.036). Similarly, as the severity of PH increased, the proportion of low-birthweight (< 2 500 g) babies born to these mothers increased (p < 0.001), again with the most low-birthweight babies born to women in the severe PH category. This was also the finding in the ROPAC study.^7^ This could be explained by the fact that the group of women with severe PH also had the highest proportion of preterm deliveries, hence the higher proportion of low-birthweight babies.

 This study is strengthened by the size of the cohort, which is large compared to previous studies on the subject. Also, all women were managed at a single centre with a uniform management protocol and all echocardiograms were performed by a single operator.

 One limitation is that our study was retrospective. Also, our cohort included only women who were managed at statesubsidised facilities and referred to IALCH, hence it does not represent the entire population of pregnant women with PH in KZN, who may be managed at private healthcare institutions, or those who may have died prior to referral to IALCH. Our follow-up period of six weeks postpartum may have limited the outcome findings as there may have been delayed outcomes beyond six weeks postpartum that our study cannot report on.

## Conclusion

Traditionally, pregnancy has been contra-indicated in women with PH. Our study supports many of the findings of other recent studies, showing decreased maternal morbidity and mortality rates and improved perinatal outcomes in subsets of women in certain aetiological categories and in those with milder forms of PH. Counselling and management practices should be individualised, considering the findings of the most recent literature. 
